# Fibroadenoma in axillary supernumerary breast: case report

**DOI:** 10.1590/S1516-31802005000500011

**Published:** 2005-09-01

**Authors:** Délio Marques Conde, Renato Zocchio Torresan, Eiji Kashimoto, Luiz Eduardo Campos de Carvalho, Cássio Cardoso

**Keywords:** Breast diseases, Fibroadenoma, Breast neoplasms, Axilla, Diagnosis, Doenças mamárias, Fibroadenoma, Neoplasias mamárias, Axila, Diagnóstico

## Abstract

**CONTEXT:**

Supernumerary breast tissue may be affected by the same diseases and alterations that compromise topical breast tissue. Nevertheless, reports of fibroadenoma in supernumerary breast tissue in the axillae are rare.

**OBJECTIVE:**

To describe a case of fibroadenoma in an axillary supernumerary breast.

**DESIGN:**

Case report.

**CASE REPORT:**

A 39-year-old woman was referred to the gynecology and obstetrics outpatient clinic at Hospital Estadual Sumaré, complaining of bilateral axillary masses. The patient reported cosmetic problems and local pain and discomfort. On physical examination, alterations compatible with bilateral axillary accessory breasts, without palpable nodules, were observed. Supplementary examinations (mammography and ultrasonography) revealed a 1.1 cm mass in the right axillary breast. The patient underwent resection of the supernumerary breasts and histopathological examination revealed fibroadenoma of the right axillary breast tissue.

## RESUMO

**CONTEXTO:**

O tecido mamário supranumerário pode ser acometido pelas mesmas doenças e alterações que comprometem o tecido mamário tópico. Porém, o relato de fibroadenoma em mama acessória axilar é raro.

**OBJETIVO:**

Descrever um caso de fibroadenoma de mama supranumerária axilar.

**TIPO DE ESTUDO:**

Relato de caso.

**RELATO DE CASO:**

Mulher de 39 anos, encaminhada ao Ambulatório de Ginecologia e Obstetrícia do Hospital Estadual Sumaré, com queixa de massa axilar bilateral. Paciente referia problemas cosméticos, dor e desconforto local. No exame físico, observaram-se alterações compatíveis com mamas acessórias axilares bilaterais, sem nódulos palpáveis. Os exames complementares (mamografia e ultra-sonografia) demonstraram nódulo de 1.1 cm em mama axilar direita. Paciente foi submetida à ressecção de mamas supranumerárias, com exame histopatológico revelando fibroadenoma de tecido mamário axilar direito.

## INTRODUCTION

Anomalies associated with breast development are not uncommon. Between 1% and 5% of women and men present supernumerary nipples (polythelia) and less often, supernumerary breasts (polymastia).^1^ These alterations are more common in women and are most frequently located along the mammary line, extending from the axilla to the pubic region. Polymastia is most commonly found in the axillae.^1,2^ Although controversial, an association between these anomalies and renal malformations has been described.^2^

Supernumerary breast tissue is subject to the same alterations and diseases, whether benign or malignant, that affect normal breast tissue.^1,2^ Since publications describing this anomaly are rare in the literature, we decided to report on a case of fibroadenoma in axillary breast tissue.

## CASE REPORT

A 39-year-old white female patient was referred to the gynecology and obstetrics outpatient clinic at Hospital Estadual Sumaré, in October 2003. She complained of masses in the axillary region, bilaterally, which were associated with cosmetic problems, local pain and discomfort, especially during her menstrual periods. She reported having noticed these changes during her first pregnancy 19 years previously, due to increased volume and pain.

Her gynecological and obstetric history was the following: menarche and thelarche began at the age of 12; she had her first child at the age of 20; she had a second pregnancy that was also carried to term; and finally she was sterilized by tubal ligation. There was no family history of breast cancer and polymastia.

On physical examination, we observed symmetrical breasts, and no masses or papillary discharge. In the axillae, there were subcutaneous masses (right mass measuring 7 cm in diameter, and left mass of 5 cm) that were painful, without palpable nodules or lymph nodes. Nipples and areola were absent. No similar alterations were found in other parts of her body.

**Figure 1:**
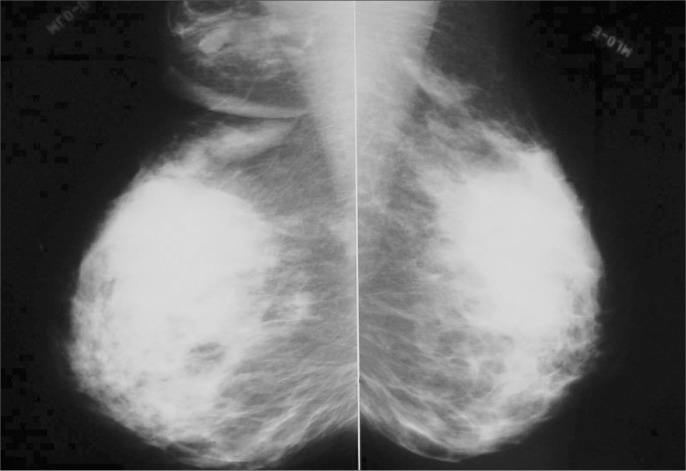
Mammogram showing round, partially circumscribed mass without calcifications, measuring 1.1 cm in diameter, in the right axillary supernumerary breast of a 39-year-old woman.

**Figure 2:**
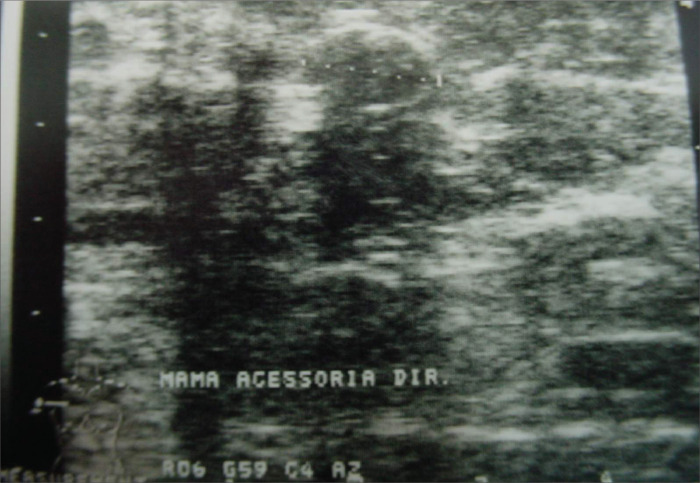
Ultrasound scan of the right axillary supernumerary breast of a 39-year-old woman showing solid, clearly circumscribed homogeneous mass of 1.1 cm diameter.

A mammogram that had been made in January 2003 did not show any abnormality that would give rise to suspicion of malignancy in bilateral supernumerary breasts. The right axillary breast exhibited a round, partially circumscribed mass, measuring 1.1 cm in diameter (Figure 1). Ultrasonography of the accessory breasts performed in November 2002 had revealed a 1.1 cm mass in the right axilla. A new ultrasound scan was performed, which demonstrated no abnormality in the left axillary breast, but showed a clearly circumscribed, homogeneous solid mass, measuring 1.1 cm in diameter in the right axilla (Figure 2), thus maintaining the same characteristics as seen previously. No biopsy or cytological study of the mass was performed. Renal ultrasonography was normal.

**Figure 3:**
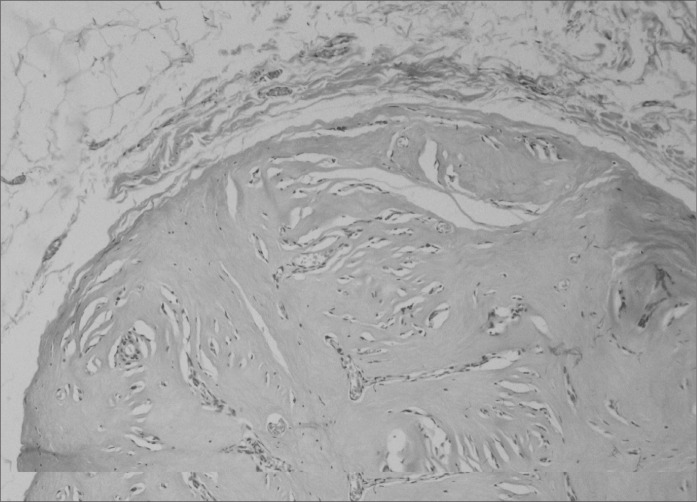
Photomicrograph (hematoxylin and eosin; 400 X) showing fibroadenoma of the right axillary supernumerary breast.

The patient underwent surgical resection of the supernumerary breasts in October 2003. Histopathological examination revealed bilateral breast tissue, with no abnormality in the left side and fibroadenoma in the right side, measuring 1.2 cm in diameter (Figure 3). The patient is currently undergoing follow-up and remains asymptomatic.

## DISCUSSION

Supernumerary breast tissue is well documented in the medical literature, and polymastia is one of its most common presentations.^1,2^ However, reports of benign and malignant tumors in supernumerary breasts are rare.^3,4^

The present case describes fibroadenoma in the axillary breast of a 39-year-old woman. The patient had been presenting complaints of bilateral axillary masses for 19 years. The progression of the fibroadenoma could not be accurately estimated. Nevertheless, on the basis of ultrasonography performed in November 2002, it could be observed that the tumor had remained static for approximately one year. Fibroadenoma is a frequent cause of nodules in young women, with highest incidence between the ages of 20 and 30 years.^1^ It is rarely described in axillary supernumerary breasts.^3^ Evidence from the natural history of fibroadenoma suggests that less than 5% of these tumors increase, whereas approximately one-fourth decrease in size.^1^ Aughsteen et al. reported a case of fibroadenoma in the axillary supernumerary breast of a 28-year-old woman, whose clinical and mammographic tests were normal. However, histopathological examination revealed fibroadenoma in the supernumerary breast.^3^

Although rare, malignant neoplasia may affect ectopic breast tissue. Pardo et al. described a case of infiltrating ductal carcinoma in super-numerary breast tissue, in a 44-year-old woman. Mammography and cytology were used for diagnostic evaluation. Therapy was based on the standard treatment used for breast cancer (surgery, chemotherapy and radiation therapy).^4^

Tumors in supernumerary breast tissue should be diagnosed with the same methods applied to normal breast tissue (mammography, ultrasonography, cytology and biopsy), observing specific indications. However, due to its low incidence, diagnosis may be delayed or even ignored, thus making treatment more difficult. When tumors or nodules are found along the mam-mary line, the presence of breast tissue should be considered during the investigation.^2^

This case demonstrates a rare occurrence of fibroadenoma in an axillary supernumerary breast. Although the benign nature and natural history of fibroadenoma are well known, biopsy should be considered for women aged 40 years or older, due to the increased rate of cancer in this age range. Among women of this age, if conservative management is chosen, periodic clinical and mammographic control is required, following negative cytological tests.

The need for careful investigation of supernumerary breast tissue should be emphasized, because it may be affected by benign and malignant diseases.
